# Supplementary material for: The zinc finger protein CLAMP promotes long-range chromatin interactions that mediate dosage compensation of the Drosophila male X-chromosome
Source: Epigenetics Chromatin. 2021 Jun 29;14:29. doi: 10.1186/s13072-021-00399-3 (PMC8240218; doi:10.1186/s13072-021-00399-3)

Jordan and Larschan Figure S1

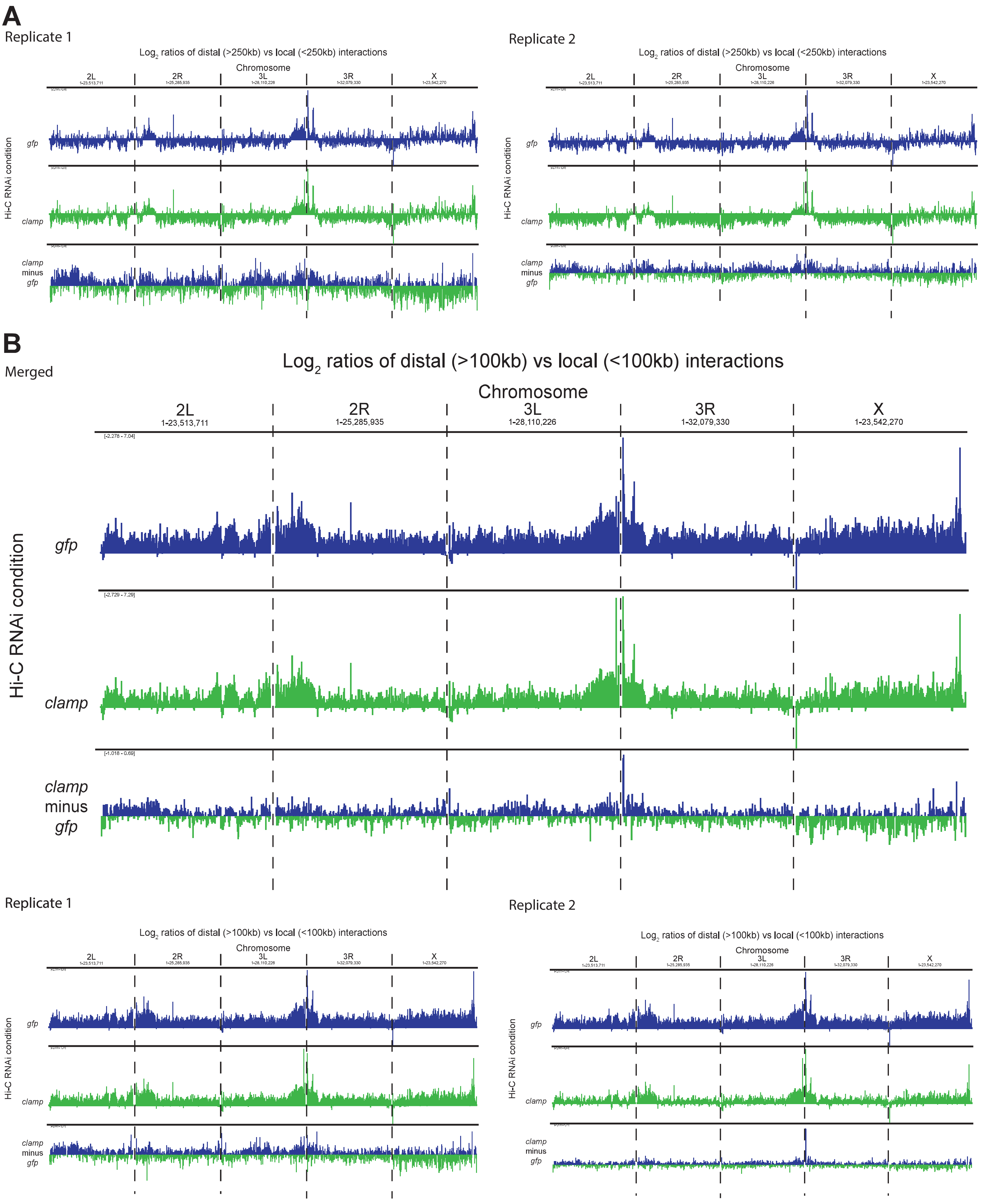

Jordan and Larschan Figure S2

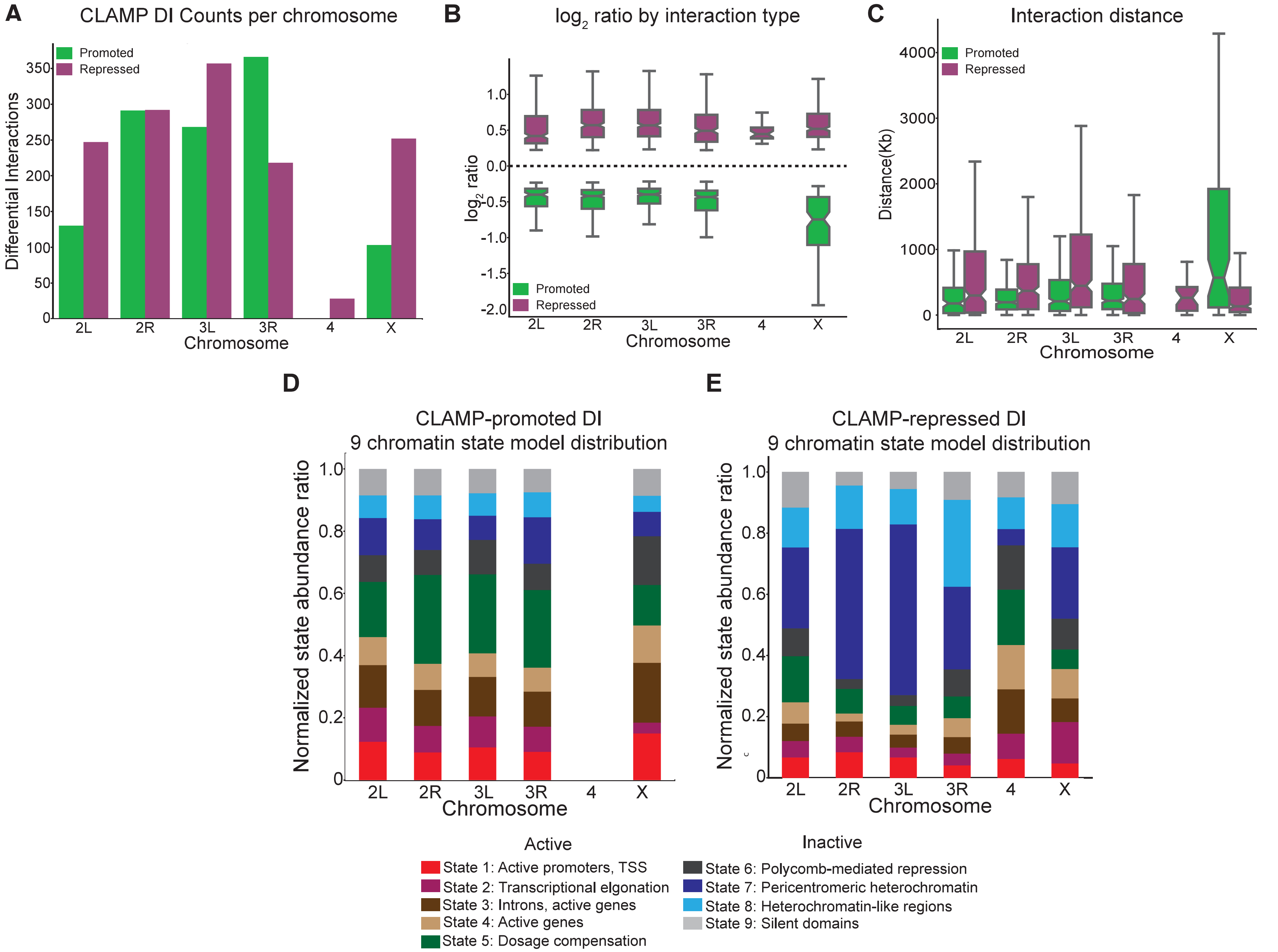

Jordan and Larschan Figure S3

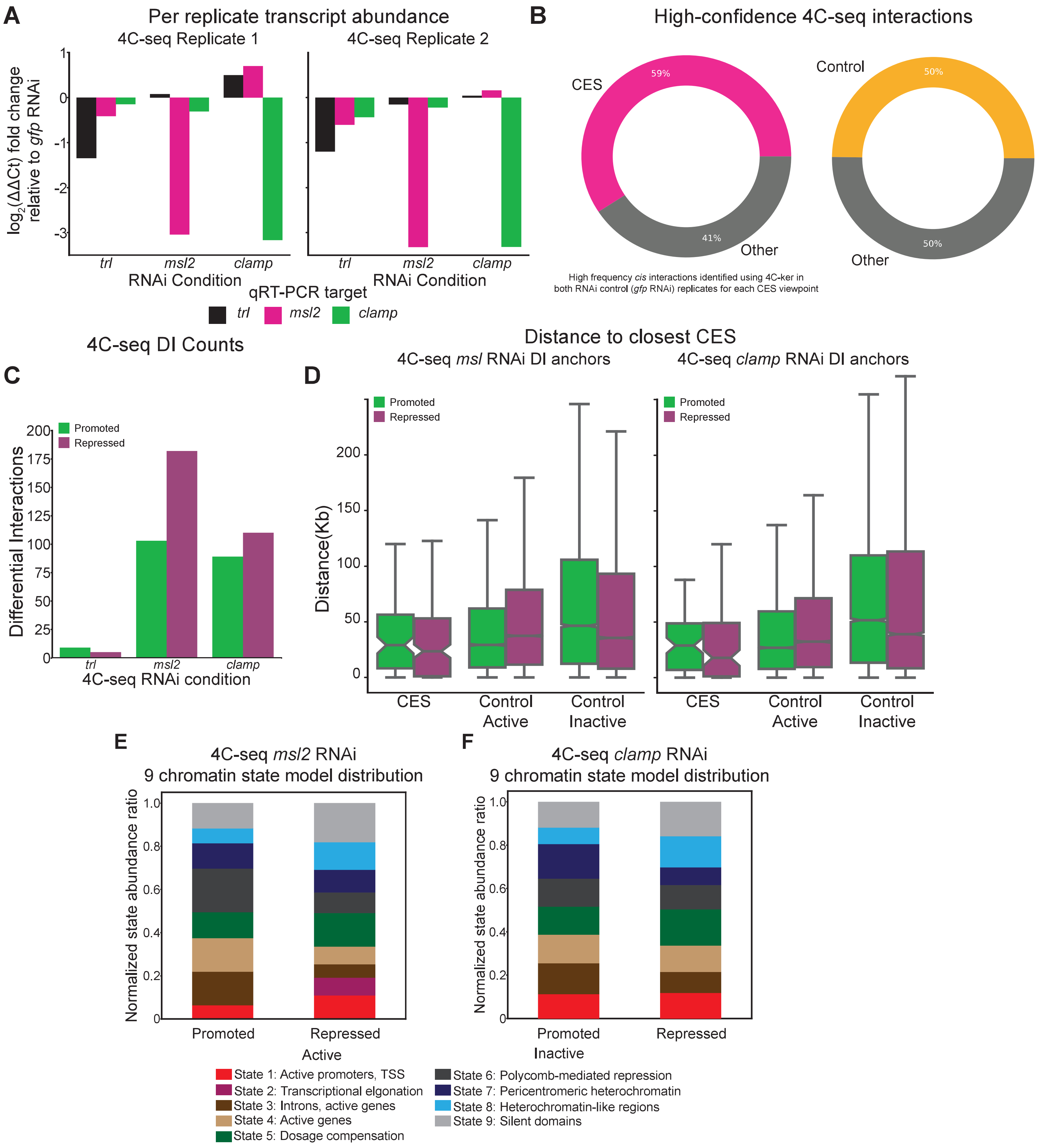

Supplement: Supplementary file 2 — Additional file 2: Fig. S1. CLAMP regulates the length span of genomic interactions on the male X-chromosome, related to Figure 2. A. Per chromosome distal vs local ratio (DLR) for gfp RNAi (blue), clamp RNAi (green), and clamp vs gfp RNAi (bottom). For the clamp vs gfp RNAi comparison, a positive number (blue) indicates the ratio of distal vs. local interactions becomes higher following clamp RNAi. A negative number (green) indicates the ratio of distal vs. local interactions becomes lower following clamp RNAi. Similar to Figure 2. but shown for paired individual replicates. B. Per chromosome (DLR) for merged (top) and paired individual replicates (bottom) using a distance > 100kb to denote distal interactions. Fig. S2. CLAMP promotes long-range interactions on the X-chromosome and generally promotes interactions in active chromatin and represses interactions in inactive chromatin. A. Per chromosome differential interaction count of CLAMP-promoted and CLAMP-repressed interactions (Source data provided in Table S4). B. Per chromosome log2 ratios by interaction type for CLAMP-promoted and CLAMP-repressed interactions, related to Figure 2A (Source data provided in Table S4). C. Per chromosome distribution of distances between differential interaction anchors for CLAMP- promoted and CLAMP-repressed interactions, related to Figure 2B (Source data provided in Table S4). D. Per chromosome normalized ratio of chromatin states occurring at CLAMP-promoted interactions (Source data provided as a Source Data file).E. Per chromosome normalized ratio of chromatin states occurring at CLAMP-repressed interactions (Source data provided as a Source Data file). For all box and whisker plots, the 95% confidence interval is shown with a notch around the median line; whiskers represent 1.5 IQR; outliers have been omitted. Fig. S3. Summary of high-resolution 4C-seq analysis. A. Quantitative real-time PCR indicates successful RNAi knockdown of each target gene. Plotted is the log2 fold ch [file 13072_2021_399_MOESM2_ESM.pdf]
